# Differential gene expression in anatomical compartments of the human eye

**DOI:** 10.1186/gb-2005-6-9-r74

**Published:** 2005-08-17

**Authors:** Jennifer J Diehn, Maximilian Diehn, Michael F Marmor, Patrick O Brown

**Affiliations:** 1 Department of Ophthalmology, Stanford University School of Medicine, Stanford, CA 94305, USA; 2 Department of Biochemistry, Stanford University School of Medicine, Stanford, CA 94305, USA; 3 Howard Hughes Medical Institute, Stanford University School of Medicine, Stanford, CA 94305, USA; 4 Department of Ophthalmology, University of California, San Francisco, San Francisco, CA 94143, USA

## Abstract

**Background:**

The human eye is composed of multiple compartments, diverse in form, function, and embryologic origin, that work in concert to provide us with our sense of sight. We set out to systematically characterize the global gene expression patterns that specify the distinctive characteristics of the various eye compartments.

**Results:**

We used DNA microarrays representing approximately 30,000 human genes to analyze gene expression in the cornea, lens, iris, ciliary body, retina, and optic nerve. The distinctive patterns of expression in each compartment could be interpreted in relation to the physiology and cellular composition of each tissue. Notably, the sets of genes selectively expressed in the retina and in the lens were particularly large and diverse. Genes with roles in immune defense, particularly complement components, were expressed at especially high levels in the anterior segment tissues. We also found consistent differences between the gene expression patterns of the macula and peripheral retina, paralleling the differences in cell layer densities between these regions. Based on the hypothesis that genes responsible for diseases that affect a particular eye compartment are likely to be selectively expressed in that compartment, we compared our gene expression signatures with genetic mapping studies to identify candidate genes for diseases affecting the cornea, lens, and retina.

**Conclusion:**

Through genome-scale gene expression profiling, we were able to discover distinct gene expression 'signatures' for each eye compartment and identified candidate disease genes that can serve as a reference database for investigating the physiology and pathophysiology of the eye.

## Abstract 2

DNA microarrays (representing approximately 30,000 human genes) were used to analyze gene expression in six different human eye compartments, revealing candidate genes for diseases affecting the cornea, lens and retina.

## Background

The human eye is composed of multiple substructures of diverse form, function, and even embryologic origin that work in concert to provide us with our sense of sight. Identifying the global patterns of gene expression that specify the distinctive characteristics of each of the various compartments of the eye is an important step towards understanding how these complex normal tissues function, and how dysfunction leads to disease. The Human Genome sequence [1,2] provides a basis for examining gene expression on a genomic scale, and cDNA microarrays provide an efficient method for analyzing the expression of thousands of genes in parallel. Previous studies have used microarrays to investigate gene expression within normal eye tissues, including cornea [3] and retina [4], as well as within pathological tissues such as glaucomatous optic nerve heads [5], uveal melanomas [6], and aging retina [7].

Analysis of gene expression in the eye has been notoriously difficult because of the technical obstacles associated with extracting sufficient quantities of high quality RNA from the tissues. This is especially true for the lens and cornea, which have relatively few RNA-producing cells when compared to a highly cellular tissue such as retina. Furthermore, pigmented ocular tissues contain melanin, which often co-purifies with RNA and inhibits subsequent enzymatic reactions [8]. Any delay between the patient's death and the harvesting of ocular tissues can also compromise RNA quality and yield. To date, many experiments examining the gene expression profile of particular eye compartments have relied on pooled samples or cell culture in order to obtain adequate amounts of RNA. In contrast to these studies, the experiments described in this paper were performed using a linear amplification procedure [9], which made it possible to examine individual specimens using DNA microarrays, thereby eliminating the potentially confounding effects of pooling multiple donor samples or culturing cells, which can elicit dramatic changes in gene expression based on the cell culture media [10]. We chose an *in vitro *transcription-based, linear amplification approach because this has previously been shown to reproducibly generate microarray gene expression results that are extremely similar to data generated using unamplified RNA [9,11,12]. Additionally, the amplification process has been shown to selectively and reproducibly 'over-amplify' some low-copy number transcripts, resulting in a larger fraction of the expressed genome that can be reliably measured on DNA microarrays. Importantly, by analyzing individual donor samples on arrays, we can detect variation in the eye compartments of different donors, which will be critical for future studies that examine how gene expression varies between individuals at baseline and also in disease states.

A major goal of this study was to discover how the various eye compartments differ from one another on a molecular level by identifying clusters of differentially expressed genes, or 'gene signatures', characteristic of each eye compartment. We also wanted to investigate how gene expression varies between geographical regions of the retina. Because certain retinal diseases such as retinitis pigmentosa (RP) and age-related macular degeneration (ARMD) preferentially affect a specific retinal region, identification of genes that are differentially expressed in the macula versus peripheral retina may provide valuable clues to the molecular mechanisms underlying these diseases. Recent work using serial analysis of gene expression (SAGE), a method that involves sequencing thousands of transcripts from a given RNA sample, identified several genes that were significantly enriched in either the macula or the periphery [13]. Our cDNA microarray studies confirmed some of these genes, but also significantly added to the catalog of macula-enriched genes. Lastly, because many ophthalmologic diseases preferentially affect a particular eye compartment, our study demonstrates that gene signatures can be combined with gene linkage studies in order to identify candidate disease genes.

## Results

**Figure 1:**
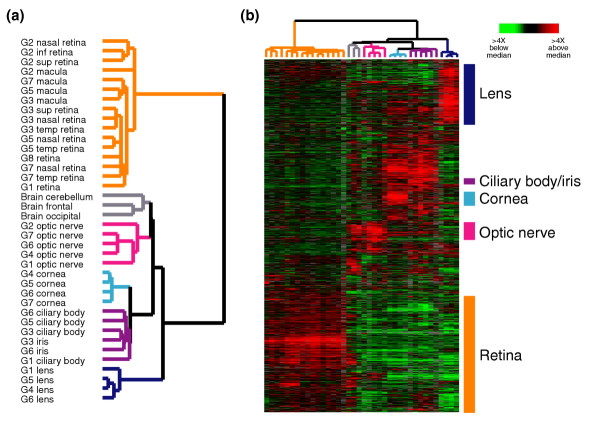
Gene expression programs in the human eye. Unsupervised hierarchical clustering of 38 samples from human cadaver eyes and normal brain. Array elements that varied at least 2.5-fold from the median on at least two microarrays were included (9,634 cDNA elements representing approximately 6,600 genes). **(a) **Array dendrogram. G1 to G8 indicate the globes from which each compartment sample was dissected (see Materials and methods). Inf., inferior; Sup., superior; Temp., temporal. **(b) **Cluster image. Data are displayed as a hierarchical cluster where rows represent genes (unique cDNA elements) and columns represent experimental samples. Colored pixels capture the magnitude of the response for any gene, where shades of red and green represent induction and repression, respectively, relative to the median for each gene. Black pixels reflect no change from the median and gray pixels represent missing data. Compartment-specific gene signatures are indicated. See our website for a searchable version of this cluster [75].

To explore relationships among the different eye compartments and among genes expressed in these compartments, we performed hierarchical cluster analysis of both genes and samples [14] using genes that met our selection criteria (see Materials and methods). The display generated through hierarchical clustering analysis is shown in Figure 1a. In this display, relatively high expression levels are indicated by a red color, and relatively low expression levels are represented by a green color; each column represents data from a single tissue sample, and each row represents the series of measurements for a single gene. Tissue samples with similar gene expression patterns are clustered adjacent to one another, and genes with similar expression patterns are clustered together. In our experiments, samples of the same eye compartment from different donors clustered in discrete groups (for example, cornea with cornea, retina with retina), with the only exception being an intermingling of the ciliary body and iris specimens (Figure 1a). The lack of a clear distinction between the expression patterns of the ciliary body and iris may be due to both their shared embryological origin and their close anatomical approximation, resulting in sub-optimal separation during dissection. The division between the retinal samples and all other samples was the most striking. Furthermore, there was a distinct grouping of the various macula specimens, which formed a tightly clustered subgroup among the retinal samples. The expression patterns of the optic nerve samples were most similar to those of the three brain specimens.

Each anatomical compartment of the eye expressed a distinct set of genes that were not expressed, or expressed at much lower levels, in the other eye compartments (Figure 1b). The repertoire of genes specifically expressed in the retina was especially large and diverse (3,727 genes), but we also found a surprisingly large number of transcripts (1,777 genes) expressed predominantly in the lens. To explore the connections between these compartment-enriched genes and phenotypic features of the compartments in which they were expressed, we considered each group of compartment-enriched genes in detail.

### Corneal signature

**Figure 2:**
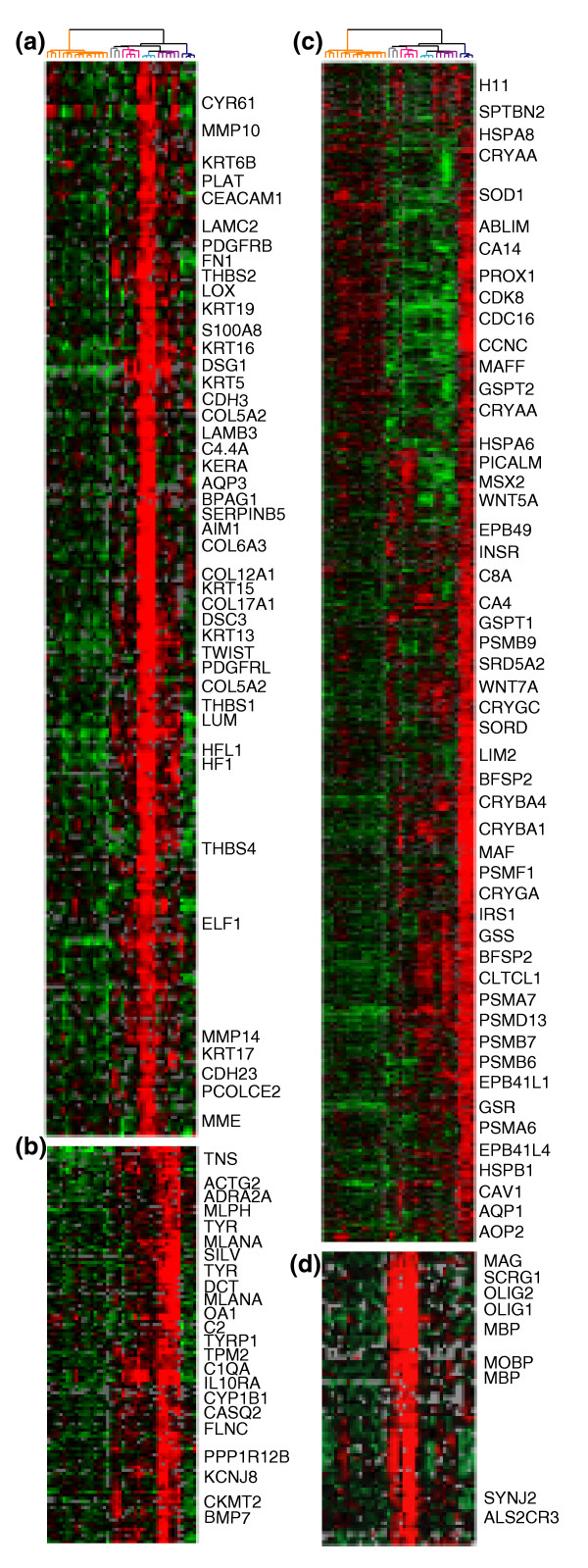
Expanded view of compartment-specific gene expression signatures in the human eye. Data were extracted from Figure 1 and are displayed similarly. Individual clusters depict genes associated with **(a) **cornea, **(b) **ciliary body and iris, **(c) **lens and **(d) **optic nerve. Many of the array elements encode uncharacterized genes and only a subset of named genes is shown.

The cornea is a multi-layered structure consisting of an epithelium of stratified squamous cells, a thick stroma of layered collagen fibrils, and an underlying endothelial layer. To provide an effective physical barrier to the outside world, the corneal epithelial cells bind to one another and to the underlying connective tissue through a series of linked structures known collectively as the 'adhesion complex'. As shown in Figure 2a, many genes enriched in the corneal signature encoded proteins that stabilize epithelial sheets and promote cell-cell adhesion, including keratins (KRT5, KRT6B, KRT13, KRT15, KRT16, KRT17, KRT19), laminins (LAMB3, LAMC2), and desmosomal components (DSG1, DSC3, BPAG1).

Other genes highly expressed in the cornea signature encoded proteins that help maintain the shape, transparency, or integrity of the cornea, which serves as the primary refractive element in the eye. Some of the genes encoded proteins specifically expressed by either squamous epithelial cells or fibroblasts, reflecting the histological composition of corneal tissue. For example, the signature included numerous genes that encode collagens (COL5A2, COL6A3, COL12A1, COL17A1), along with the gene for lysyl oxidase (LOX), an enzyme that promotes collagen cross-linking. The gene encoding keratocan (KERA), a proteoglycan involved in maintaining corneal shape in mice knock-out studies [15], and linked to abnormal corneal morphology (keratoconus and cornea plana) in humans, was selectively expressed in corneal tissue, as were the genes encoding lumican (LUM), a keratan sulfate-containing proteoglycan that has been shown to be important for mouse corneal transparency [16], and aquaporin 3 (AQP3), which encodes a water/small solute-transporting molecule. Immunolabeling studies performed on corneas with pseudophakic bullous keratopathy demonstrated increased AQP3 in the superficial epithelial cells, suggesting that AQP3 may be associated with increased fluid accumulation, resulting in the decrease in corneal transparency seen in pseudophakic bullous keratopathy corneas [17]. Modulating genes or proteins involved in corneal shape and transparency could potentially lead to non-invasive treatments for some corneal diseases, which are often only remediable through corneal transplantation.

An intriguing subset of genes in the cornea signature has been studied in tumor metastasis models because these genes encode proteins that regulate cell-cell or cell-matrix interactions (TWIST, MMP10, SERPINB5, THBS1, CEACAM1, C4.4A). For example, TWIST encodes a transcription factor shown to promote metastasis in a murine breast tumor model through the loss of cadherin-mediated cell-cell adhesion [18]. Another corneal signature gene encodes matrix metalloproteinase 10 (MMP10), a protein capable of degrading extracellular matrix components. Overexpression of MMP10 in transfected lymphoma cells has been shown to stimulate invasive activity *in vitro *and promote thymic lymphoma growth in an *in vivo *murine model [19]. Various matrix metalloproteinases have been examined for their roles in corneal wound healing (reviewed in [20]), including MMP10, which was identified in migrating epithelial cells in cultured human cornea tissues that were experimentally wounded [21], which may suggest that the process of corneal wound healing may mimic some aspects of tumor biology. Certainly, in both wound healing and cancer, cells undergo rapid proliferation, invade and remodel the extracellular matrix, and migrate to other areas.

Recent microarray investigations identified a gene expression signature related to a wound response in the expression profiles of several common carcinomas, and the presence of this wound healing gene signature predicted an increased risk of metastasis and death in breast, lung, and gastric carcinomas [22,23]. Further research into corneal wound healing may also provide us with a model for better understanding the pathophysiology underlying tumor metastasis because the cornea is exceptionally efficient among human tissues at degrading and remodeling its extracellular matrix, allowing it to heal superficial wounds within hours.

### Ciliary body/iris signature

The ciliary body and iris are components of the eye's highly pigmented and vascular layer known as the uveal tract. As might be expected, genes related to pigmentation were a feature of the distinctive expression pattern of these tissues (Figure 2b). These genes encoded enzymes involved in melanogenesis, including tyrosinase (TYR), tyrosinase-related protein 1 (TYRP1), and dopachrome tautomerase (DCT), as well as melanosomal matrix proteins such as SILV and MLANA. Several of the ciliary body/iris signature genes were noteworthy in that their mutation can lead to albinism or hypopigmentation phenotypes, including OA1 (ocular albinism type 1), TYR and TYRP1 (oculocutaneous albinism 1A and 3, respectively), and MLPH (Griscelli syndrome). Investigation of the numerous uncharacterized genes with similar expression patterns to those of pigmentation genes may expand our knowledge about the pigmentation process in eyes and the molecular mechanisms behind hypopigmentation syndromes.

The ciliary body is also responsible for aqueous humor formation and lens accommodation, while the contiguous iris filters light entering the eye by constricting and dilating the muscles around the pupillary opening. Histologically, the ciliary body consists predominately of smooth muscle, but also contains striated muscle (reviewed in [24]). Previous work has demonstrated that contractility of both the ciliary body and the trabecular meshwork is critical in modulating aqueous humor outflow (reviewed in [25]), one of the key determinants of intraocular pressure, along with aqueous humor production and episcleral venous pressure. Muscle-related proteins encoded by genes in the ciliary body/iris cluster included smooth muscle actin (ACTG2), and actin cross-linking proteins such as filamin (FLNC), tropomyosin (TPM2), and tensin (TNS). Other iris/ciliary body signature genes have known roles in myosin phosphorylation (PPP1R12B), sarcolemmal calcium homeostasis (CASQ2), and ATP availability (CKMT2), all of which may contribute to ciliary body/trabecular meshwork contractility.

Both ciliary body and trabecular meshwork contractility, as well as aqueous humor production, have been linked to changes in membrane potential, and membrane channels have been studied extensively in the ciliary body [25-27]. Of note, transcripts encoding an inward-rectifying potassium channel (KCNJ8), not previously identified in the ciliary body, were highly enriched in the ciliary body/iris signature and may warrant further study. The signature also included the gene for adrenergic receptor 2α (ADRA2A), a regulator of aqueous humor production and outflow, and the molecular target of the ocular hypotensive agent brimonidine. Identification of other genes that facilitate aqueous production and outflow may provide additional molecular targets for future glaucoma therapeutics aimed at lowering intraocular pressure, the only modifiable risk factor for the development and progression of glaucoma.

### Immune system genes expressed within anterior segment tissues

Genes related to immune defense mechanisms were prominent among the large set of genes selectively expressed in both the ciliary body/iris and corneal tissues. These included genes encoding proteins involved in intracellular antigen processing and transport for eventual surface presentation to immune cells (PSMB8, TAP1), antigen presentation proteins, including HLA class I molecules (HLA-A, HLA-C, HLA-F, and HLA-G) and HLA class II molecules (HLA-DRB1, DRB4, DRB5, DPA1, and DPB1), cytokines involved in the recruitment of monocytes (SCYA3, SCYA4, CD14), and cytokine receptors (IL1R2, IL4R, and IL6R). Several anterior segment-enriched genes encoded proteins with intrinsic antibiotic activity, including defensin (DEFB1) and lysozyme (LYZ), which may protect epithelial surfaces from microbial colonization.

Genes encoding components of the complement cascade, a major arm of the innate immune system, were a particularly prominent feature of the anterior segment signature. Most of the early classical pathway complement genes, including C1 components (C1S, C1QA, C1QG, C1R), C2, and C4b, as well as a component of the late complement cascade (C7), were selectively expressed in both the corneal and ciliary body/iris tissues. In addition, the gene encoding the trigger for the alternative complement pathway, properdin (BF), was highly expressed in these tissues.

To prevent the destructive reactions that could ensue from the daily bombardment of the eye with potentially antigenic stimuli, regulatory mechanisms must counteract the multitude of pro-inflammatory mediators found in the eye. A study by Sohn *et al*. [28] that examined a number of complement and complement-regulating components in rat eyes suggested that the complement system is continuously active at a low level in the normal eye and is kept in check by regulatory proteins. Indeed, we found that the anterior segment selectively expressed many critical negative regulators of the immune system, especially of the complement cascade. These included SERPING1 and DAF, two genes that encode proteins that limit the production of early complement components, and CD59, which encodes a protein that inhibits the assembly of complement subunits into the membrane attack complex.

The presence of complement activation products in the human eye during infection or inflammation has been previously described [29]. Studies have suggested that the complement pathway contributes to the pathophysiology of uveitis, an inflammatory disease of the uveal tract that is often idiopathic in etiology [30]. In support of this theory, Bardenstein *et al*. [31] showed that blocking the complement regulator CD59 in the rat eye precipitated massive inflammation in the anterior eye, including intense conjunctival inflammation and iritis. Our evidence that complement pathway components and regulators are highly expressed in anterior segment tissues provides further impetus for investigating their links to ocular disease.

A caution to bear in mind in interpreting these results is that all of our ocular specimens were obtained post-mortem. The expression of the inflammatory genes could therefore reflect, at least in part, changes in the eye that occur after death. Future studies examining gene expression in fresh tissue samples obtained at surgery, such as peripheral iridectomy specimens, should help to further address this issue.

### Lens signature

The distinctive features of the lens are its transparency, precisely crafted shape, and deformability, all of which are critical for proper light refraction. Elucidating the molecular mechanisms that maintain or disrupt lens transparency is fundamental in preventing cataract, the leading cause of world blindness. Our studies showed that lens gene expression is very distinct from the other eye compartments (Figure 2c), perhaps reflecting the extraordinary specialization of the lens as an isolated, avascular structure within the eye. We found more than a thousand genes selectively expressed in the lens; clearly, diverse RNA populations are still present in the adult lens, even though its population of active epithelial cells is outnumbered by the mature fiber cells that have lost their organelles, including nuclei.

Genes encoding the subunits of crystallins, the predominant structural proteins in the lens, were prominent in the lens signature, including subunits for crystallin alpha (CRYAA), beta (CRYBA1, CRYBA4), and gamma (CRYGA, CRYGC). Work by Horwitz and colleagues [32,33] on alpha-crystallins, which are structurally similar to small heat shock proteins, showed these crystallins may preserve lens transparency by serving as molecular chaperones that protect other lens proteins from irreversible denaturation and aggregation. Of the other heat shock proteins highly enriched in the lens signature (HSPA6, HSPA8, HSPB1), HSPB1 may be of particular interest because it is a protein with an alpha-crystallin domain that may have a role in lens differentiation [34]. The lens signature also included genes encoding subunits of the proteasome complex (PSMA6, PSMA7, PSMB6, PSMB7, PSMB9, PSMD13), a multicatalytic proteinase structure that is responsible for degrading intracellular proteins. Previous studies have demonstrated the significance of the proteasome pathway in removing oxidatively damaged proteins within the lens [35].

Besides the crystallin genes, other genes encoding previously described structural components of the lens, including lens intrinsic membrane (LIM2), beaded filament structural protein (BFSP2), spectrin (SPTBN2), and actin binding protein (ABLIM) were included in the lens signature. More interestingly, the signature also contained intermediate filament genes, such as those encoding erythrocyte membrane band 4.9 and 4.1 (EPB49 and EPB41L1, EPB41L4), that are characteristically expressed in erythrocytes, another cell whose highly stereotyped shape is critical to its function. Previous studies have shown that protein 4.1 helps stabilize the spectrin-actin cytoskeleton, which is present in both erythrocytes and lenticular tissue [36]. Further investigations comparing erythroid and lens cells may reveal other similarities in their cytoskeletons, both of which define a distinctive and stereotyped cell shape that must endure substantial amounts of mechanical stress.

Another notable feature of the lens signature was the enrichment of genes encoding proteins involved in endocytosis, including clathrin (CLTCL1, PICALM) and caveolin (CAV1). Currently, intercellular transport within the lens is thought to occur predominately by diffusion through gap junctions, but several investigators have proposed the uptake of nutrients must be supplemented by mechanisms other than gap junctions because of the paucity of gap junctions identified in microscopy studies and the confirmed presence of clathrin-coated vesicles in freeze-fracture studies [37,38].

Oxidative stress mediated by free radical production has been associated with cataract formation (reviewed in [39]). Therefore, we looked for genes involved in scavenging free radicals in the lens signature. Two of these genes encode enzymes, glutathione synthetase (GSS) and glutathione reductase (GSR), that facilitate the production of glutathione, a potent anti-oxidant and essential cofactor for redox enzymes. Superoxide dismutase (SOD1) and anti-oxidant protein 2 (AOP2), two proteins responsible for reducing free oxygen radicals and hydrogen peroxide species, respectively, were also selectively expressed in lens tissue. Drugs or environmental agents that modulate the expression or activity of these proteins could have a significant impact on cataract progression or prevention.

### Optic nerve signature

The gene expression pattern in the optic nerve was overall quite similar to that seen in brain tissue (Figure 2d), very likely reflecting the preponderance of glial cells present in both tissues. Both signatures included a number of genes (MBP, MOBP, MAG, OLIG1, and OLIG2) previously found in glial cells, several of which have been linked to neurological diseases. For example, myelin-associated oligodendrocyte basic protein (MOBP) is implicated as an antigen stimulus for multiple sclerosis, a disease that also can present with optic neuritis (reviewed in [40]). Interestingly, the optic nerves in MOBP knock-out mice lacked the radial component of myelination [41]. In another study, transgenic mice with T-cell receptors specific to myelin associated glycoprotein (MAG) spontaneously presented with optic neuritis [42]. The majority of the genes in the brain and optic nerve signatures encoded proteins of unknown function; our results, showing that these genes may have specialized roles in these tissues, may be a step toward discovering the biological role(s) for these uncharacterized proteins.

### Retina signature

**Figure 3:**
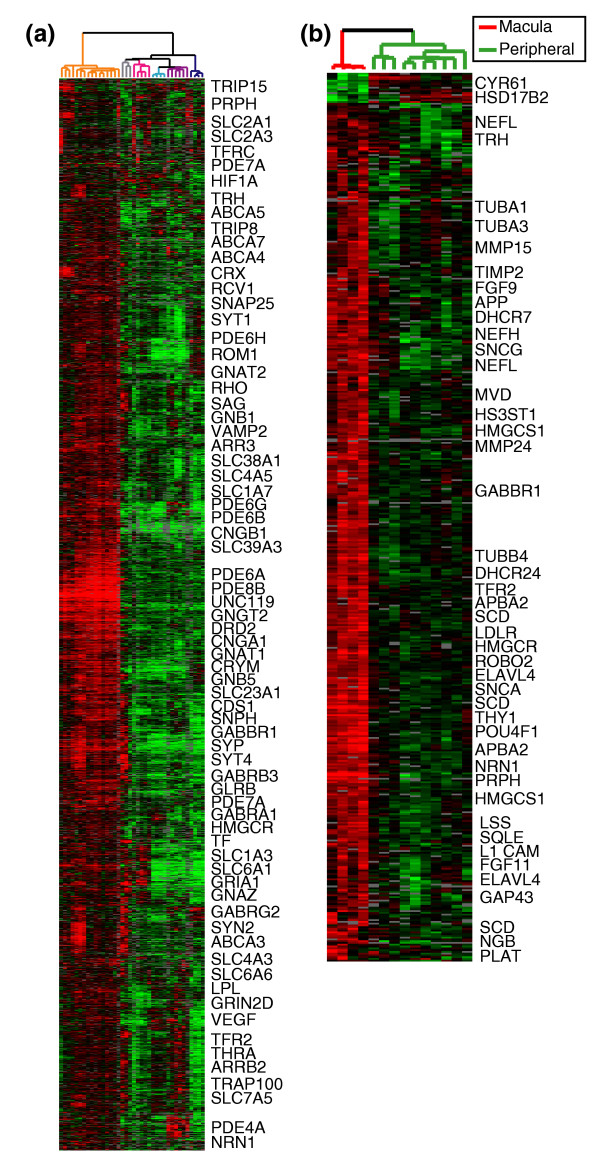
Retinal gene expression. **(a) **The retina-specific gene expression signature was extracted from Figure 1 and is displayed similarly. Many of the array elements encode uncharacterized genes and only a subset of named genes is shown. **(b) **Macula versus peripheral retina gene expression. Using the statistical analysis of microarrays algorithm as described in Materials and methods, we selected genes that differed significantly between the central and peripheral retinal arrays at a false discovery rate <0.05.

The retina, a complex tissue composed of neuronal and glial elements, is essentially an extension of the central nervous system, and the genes found in the retina signature appear to reflect its distinctive histology and embryology (Figure 3a). For example, the signature included the receptors for known retinal neurotransmitters, including gamma-aminobutyric acid (GABRA1, GABRG2, GABRB3), glutamate (GRIA1, GRIN2D), glycine (GLRB), and dopamine (DRD2). Retinal neurotransmitters are packaged into small vesicles in the pre-synaptic regions of photoreceptors. Many retinal signature genes encoded proteins associated with synaptic vesicle docking and fusion (SNAP25, VAMP2, SYP, SNPH), as well as vesicle exocytosis and neurotransmitter release (SYN2, SYT4). One of the retinal signature genes with a role in synaptic transmission, human retinal gene 4 (HRG4/UNC119), has been linked to late-onset cone-rod dystrophy in humans and marked synaptic degeneration in a transgenic mouse model [43].

The retina protects the integrity of its neuronal layers by regulating its extracellular environment through a blood-retina barrier consisting of vessel tight junctions and cell basement membranes. The exchange of nutrients and metabolites across these barriers likely requires diverse, specialized transporters. Indeed, over 30 different genes encoding small molecule transporters were found within the retina signature, including carriers of glucose (SLC2A1, SLC2A3), glutamate (SLC1A7), and other amino acids (SLC7A5, SLC38A1, SLC6A6). Of note, severe retinal degeneration was observed in mice mutated in *SLC6A6*, a gene encoding a transporter of the amino acid taurine [44]. Several genes encoding ABC transporters (ABCA3, ABCA4, ABCA5, ABCA7), which use ATP energy to transport various molecules across cell membranes, were contained in the retinal signature. The most notable of these, ABCA4, is involved in vitamin A transport in photoreceptor cells; mutations in the gene encoding ABCA4 can result in a spectrum of retinopathies, including retinitis pigmentosa, Stargardt's disease, cone-rod dystrophy, and ARMD.

The retinal signature was also enriched in transcripts encoding vitamin and mineral transporters. The inclusion of a vitamin C transporter (SLC23A1) and a zinc transporter (SLC39A3) within the signature was of particular interest, in light of the Age-Related Eye Disease Research Group study that demonstrated supplementation with zinc and anti-oxidants, including vitamin C, lowered the probability of developing neovascular ARMD in some high-risk patient subgroups [45]. The presence of transferrin (TF), an iron transport molecule, and its receptor (TFRC), in the retina signature may also be noteworthy because a higher accumulation of iron has been observed in some ARMD-affected maculas [46].

Somewhat unexpectedly, the retina signature contained the gene encoding thyroid releasing hormone (TRH) and numerous thyroid hormone receptor-related genes (THRA, TRIP8, TRIP15, TRAP100). TRH expression was previously observed in the retinal amacrine cells of amphibians [47]. Previous work has demonstrated the importance of thyroid hormone in the developing rat retina [48], and thyroid hormone receptors are required for green cone photoreceptor development in rodents [49]. Further studies of these genes may uncover additional roles of thyroid hormone and its receptors in the human retina.

The retina is ultimately responsible for executing the visual cycle, the process by which a photon signal is translated into an electrical impulse. This complex cycle is initiated when photoreceptor pigments activate G-proteins. G-proteins in turn activate phosphodiesterases to break down cyclic GMP (cGMP) to GMP, thereby influencing cell polarization via the downstream modulation of ion channel efflux. The retina signature incorporated many genes encoding known visual cycle elements, including the photopigment rhodopsin (RHO), G-proteins from rods and cones (GNAT1, GNAT2, GNB5), subunits of rod and cone phosphodiesterases (PDE6A, PDE6B, PDE6G, PDE6H), and cGMP-sensitive channels (CNGB1, CNGA1). Genes responsible for visual cycle recovery, such as arrestins (SAG, ARR3), were also present. Intriguingly, transcripts encoding other G-proteins (GNB1, GNAZ) and several phosphodiesterases (PDE8B, PDE7A, PDE4A) with no established roles in the visual cycle were enriched in the retinal signature. Additionally, the signature contained CDS1, which, though it has no clear function in humans, is homologous to the phototransduction gene CDS that has been linked to light-induced retinal degeneration in *Drosophila *mutants [50]. Perhaps further in-depth study of the many uncharacterized genes in the retinal signature will reveal roles in phototransduction for these genes, which may expand our current concept of the visual cycle pathway.

### Macula signature

We used the statistical analysis of microarrays (SAM) algorithm to select genes whose expression differed significantly between the central and peripheral retinal tissues (Figure 3b). The large set of genes that we identified as selectively expressed in macula tissues included a subset of genes involved in lipid biosynthesis. The majority of these genes are regulated by sterol response element-binding protein (SREBP), a transcription factor that has emerged as a master regulator of cholesterol and fatty acid metabolic pathways [51]. Previous studies by Fliesler *et al*. [52] have provided evidence for rapid *de novo *synthesis of cholesterol in the rat retina *in vivo*, and our findings strongly suggested the human retina also contains the enzymes needed for cholesterol biogenesis. Transcripts encoding the enzymes that catalyze multiple steps in cholesterol synthesis were enriched in the macula, including stearoyl-CoA desaturase (SCD), mevalonate decarboxylase (MVD), hydroxy-3-methylglutaryl-coenzyme A synthase 1 (HMGCS1), and HMG-coenzyme A reductase (HMGCR), the rate-limiting enzyme in cholesterol synthesis and the target of the 'statin' class of drugs for patients with dyslipidemia. Other macula signature genes encoded enzymes that act later in cholesterol biosynthesis, such as lanosterol synthase (LSS) and squalene epoxide (SQLE). In addition, the macula-enriched cluster included the gene for low-density lipoprotein receptor (LDLR), known for its role in binding low-density lipoprotein (LDL), the major cholesterol-carrying lipoprotein of plasma. LDL receptors and LDL-like receptors have been previously identified in retinal pigment epithelium and retinal muller cells [53,54], but their function in cholesterol transport within the retina has been minimally explored.

The genes represented in the macula cluster at least partially reflect cell types present in a higher density in the macula than in the peripheral retina, such as ganglion cells and photoreceptors. For example, a substantial number of genes in the macula signature have previously been characterized in ganglion cells (THY1, POU4F1, L1CAML1, NRN1). Interestingly, cholesterol is involved in the physiology of both retinal ganglion cells and photoreceptors. Cholesterol has been identified in rod outer segments in a wide variety of animal species (reviewed in [55]), as well as in oil droplets isolated from chicken cone photoreceptors [56]. *In vitro*, cholesterol has the capacity to modulate phototransduction in rods by altering the rod outer segment membrane structure [57], as well as by directly binding to rhodopsin itself [58]. Histological studies on retinas from patients with abetalipoproteinemia and familial hypobetalipoproteinemia, (serum LDL-cholesterol levels <5% of normal) demonstrated a profound absence of photoreceptors throughout most of the posterior retina [59,60]. In addition, patients with Smith-Lemli-Opitz Syndrome, a disease of abnormal cholesterol metabolism caused by a defect in 7-dehydrocholesterol reductase (DHCR7), another enzyme encoded by a gene selectively expressed in macula tissues, exhibited slower activation and recovery kinetics of their rod photoreceptors [61].

*In vitro *studies by Mauch *et al*. [62] have demonstrated that retinal ganglion cells require cholesterol in order to form mature, functioning synapses. The retinal ganglion cells in their experiments produced enough cholesterol to survive and grow, but effective synaptogenesis demanded additional cholesterol supplied by glial cells. Other work by Hayashi *et al*. [63] showed that exposure to lipoproteins containing cholesterol and apolipoprotein E stimulated retinal ganglion cell axons to extend, and that this effect was mediated by receptors of the LDL receptor family present on distal axons. Studying the role of cholesterol in synaptogenesis may lead to insights useful in the development of protective or restorative therapeutics for neurodegenerative disease, as well as for ocular diseases that affect ganglion cells.

In view of epidemiological studies that have suggested connections among atherosclerosis, serum cholesterol levels, and ARMD [64-66], the enrichment of cholesterol biosynthesis genes within the macula warrants further investigation. The presence of cholesterol in drusen, the extracellular deposits of ARMD, has been confirmed [67,68], although the origin of this cholesterol remains unclear. Disregulation of lipid metabolism and transport, either on a local and/or systemic level, may contribute to macular diseases, such as ARMD. Studies have associated statin use with a decreased rate of ARMD [69,70], but randomized, prospective studies have yet to be completed.

### Identifying candidate disease genes

One direct application of the gene expression patterns we have defined is the identification of candidate genes for genetic diseases that differentially affect the various eye compartments. This strategy relies on the hypothesis that if mutations in a gene cause physiological aberrations specifically in a particular tissue, the gene is more likely to be selectively expressed in that tissue. We therefore used the literature, RetNet [71], and the Online Mendelian Inheritance in Man [72] databases, to collate lists of genetic diseases affecting the lens, cornea, and retina, along with the genetic intervals to which the disease loci have been mapped. Next, we identified genes that were relatively selectively expressed in each of the three compartments. Briefly, we standardized the Cy5 intensity data for each array and calculated the average intensity for every gene across all samples from each compartment. We then empirically identified an intensity cut-off that resulted in selection of greater than 85% of genes included in the retinal compartment signature from Figure 1, but also included highly expressed genes that were expressed in more than one compartment. Using this cut-off, we identified separate compartment gene lists for the three compartments and identified the subset of these genes that were located in the appropriate cytogenetic intervals for each compartment-specific disease (see Additional data files 4, 5, 6 and Materials and methods).

To assess the potential of this approach, we analyzed the subset of diseases for which candidate intervals were listed in our sources but for which the causative gene is now known. The density of affected-tissue-expressed genes located in the candidate intervals was similar to that for the unknown diseases, and thus this subset served as a reliable positive control. The disease gene for a remarkable 50% to 70% of the diseases of known genetic cause was selectively expressed in the cognate compartment (Table 1). We tested the statistical significance of this result by comparing the number of disease genes identified by the compartment gene expression lists with the aggregate list of all genes detectably expressed in any of the samples shown in Figure 1. We found that for all three groups of diseases, the compartment signatures were significantly enriched for candidate disease genes (lens, p < 0.002; cornea, p < 0.005; retina, p < 0.0004, by the hypergeometric distribution). By focusing on the genes expressed within the compartment displaying the disease phenotype, we could enrich for potential candidate genes by an average of 2 to 2.5-fold.

As an example of this approach, we more closely examined Retinitis Pigmentosa 29 (RP 29), an autosomal recessive form of RP that was mapped to chromosomal region 4q32-q34 in a consanguineous Pakistani family [73]. At least two genes within this interval (*WDR17*, *GPM6A*), and one gene near the interval (*CCN3*), were previously examined by sequencing and were excluded as candidates [74]. In our data, only one gene, *KIAA1712*, was both located within the mapped interval and selectively expressed in our retinal samples. Little is currently known about this gene, except that it appears in expressed sequence tags (EST) and SAGE libraries from several tissues, including brain. Our analysis suggests that *KIAA1712 *is a strong candidate gene for RP 29, and deserves further study. We expect our candidate gene lists to be highly enriched for the causative genes for a large fraction of the diseases we analyzed, and thus should prove useful in accelerating identification of genes important in various aspects of ocular pathology.

## Discussion

Our microarray studies identified distinct molecular signatures for each compartment of the human eye. As we predicted, many of the genes differentially expressed in each tissue could be related to the histology and embryology of the cognate structure in the eye; more usefully, each signature uncovered numerous genes whose expression or function in the eye had not been previously characterized and for which their expression pattern now provides a new clue to their roles. Through a comparative analysis of gene expression among eye compartments, we can also gain insight into the pathophysiology of diseases that afflict specific eye tissues. Furthermore, our data may help anticipate or understand drug effects and side-effects, when the molecular targets of the drugs are preferentially expressed in particular ocular tissues.

The extensive set of genes selectively expressed in the macula demonstrates that there is significant regional variation in gene expression programs in the human retina. The macula-enriched expression pattern may provide clues to the pathogenesis of retinal diseases that preferentially affect the macula, such as ARMD. Because no ophthalmologic clinical data accompanied the autopsy globe samples used in our experiments and because of our limited sample sizes, we were unable to correlate our gene expression data with clinical exam findings or disease course. The techniques used in these experiments did, however, allow us to examine tissues from individual donors rather than requiring us to rely on either pooled tissue samples or cultured cells. Thus, our results show that future experiments examining individual diseased samples will be possible.

By analyzing our global gene expression data together with previous genetic mapping data, we were able to greatly refine sets of candidate genes for many corneal, lenticular, and retinal diseases whose genetic basis is still undefined. When we used a control set of diseases with known causative genes, the candidate gene lists we generated included 50% to 70% of the causative genes for this control set. One explanation for why we did not identify all the causative genes for the control disease set was that some causative genes did not meet our intensity threshold, and thus were not included in the compartment expression lists. Furthermore, we could not have identified those causative genes that are only expressed in the diseased state (but not in normal tissues), because we limited our microarray analyses to tissues with no known ocular pathology. Other reasons why our approach may have missed causative genes include expression of causative genes only at certain points in development and not in adult tissues, technical problems with the array element(s) representing these genes, and possible loss of transcripts in the RNA isolation or amplification process. Future investigation of these potential problems and comparison of our candidate gene lists with genome-scale gene expression data from diseased tissues will result in further refinement of the approach presented here.

Finally, our studies were designed to provide an open resource for all investigators interested in ocular physiology and disease. The tissue signature data, as well as the diseases, genetic intervals, and candidate genes for all the diseases we examined, and the complete set of data from our studies is freely available without restriction from the Authors' Web Supplement accompanying this manuscript [75].

## Materials and methods

### Tissue specimens

Eight whole globes (G1 to G8) were harvested from autopsy donors (age range 30 to 85 years old) within 24 h of death, and the tissues were immediately stored at 4°C in RNAlater (Ambion, Austin, TX, USA). Four of the globes were from female donors (G3, G6 to G8) and four were from male donors (G1, G2, G4, G5). Globes 4 and 5 were harvested as a set from a single donor, as were globes 6 and 7. No ophthalmologic clinical records were available for any of the globes at the time of harvest. Seven of the globes (G1 to G7) were dissected into the following components: cornea, lens, iris, ciliary body, retina, and optic nerve, while only retinal tissue was available from G8. The maculas and the peripheral retinal tissues were further dissected from several of the retinal samples. The macula was defined as the visible xanthophyll-containing tissue temporal to the optic nerve, which encompassed an approximate area of 4 mm^2^. For comparison purposes, three post-mortem brain specimens were analyzed.

### RNA extraction and amplification

Specimens were disrupted in TRIZOL (Gibco, Carlsbad, CA, USA) solution using a tissue homogenizer. Samples were processed according to the manufacturer's protocol until the aqueous supernatant was retrieved. The supernatant was mixed with 1 volume of 70% ethanol, applied to an RNeasy column (Qiagen, Valencia, CA, USA), and purified according to the manufacturer's protocol. RNA quality and quantity were assessed by gel electrophoresis and spectrophotometer measurements. Total RNA was amplified using a single round, linear amplification method [9] (also see Additional data files 1 and 2). Tissue samples that yielded inadequate amounts of RNA were excluded from any further analysis. A reference mixture of mRNAs derived from 10 different cell lines (Universal Human Reference RNA, Stratagene, La Jolla, CA, USA) was used in all experiments as an internal standard for comparative two-color fluorescence hybridization.

### Microarray procedures

Human cDNA microarray construction and hybridization were as previously described [76]. The microarrays contained 43,198 elements, representing approximately 30,000 genes (estimated by UniGene clusters) and were manufactured by the Stanford Functional Genomics Facility [77]. In each analysis, amplified RNA from an eye tissue sample was labeled with Cy5, and amplified reference RNA was labeled with Cy3. The two labeled samples were combined, and the mixture was hybridized to a microarray. Arrays were scanned using a GenePix 4000B scanner (Axon Instruments Inc., Sunnyvale, CA, USA). The array images were processed using GenePix Pro 3.0, and the resulting data were indexed in the Stanford Microarray Database and normalized using their default total intensity normalization algorithm (more detailed methods are available in Additional data file 3). Searchable figures and all raw microarray data can be found at [75]. The complete microarray dataset is also accessible through the Gene Expression Omnibus [78] (accession number GSE3023).

### Bioinformatic analyses

For the data shown in Figures 1 and 2, only elements for which at least 50% of the measurements across all samples had fluorescence intensity in either channel at least 3.25-fold over background intensity were included. The logarithm of the ratio of background-subtracted Cy5 fluorescence to background-subtracted Cy3 fluorescence was calculated. Then values for each array and each gene were median centered, and only cDNA array elements for which at least two measurements differed by more than 2.5-fold from the median were included in subsequent analyses. For the data in Figures 3, we employed the Statistical Analysis of Microarrays (SAM) package [79]. Only elements for which the intensity to background ratio was at least 3.25 in at least 35% of the retina samples were considered. Only genes whose expression significantly differed between the macula and peripheral retina (false discovery rate <0.05 with 500 permutations) were selected. Finally, to focus on genes with the largest absolute difference in expression between the two regions, we selected genes whose expression differed by at least four-fold from the median in at least two samples.

### Candidate disease gene analysis

To identify the gene sets expressed in each compartment, background-subtracted Cy5 intensities from each microarray were standardized to an array-median of 1,500, and genes exhibiting an average intensity of at least 2,500 in a compartment were identified (see Additional data file 4). This threshold was chosen empirically because it resulted in greater than 85% of the retinal signature from Figures 1 to be included in the retina set, while less than 5% of these genes were contained in any of the other compartment sets. Genetic diseases affecting the lens, cornea, or retina were collated from the Online Mendelian Inheritance in Man database [72] and the Retinal Information Network [71], along with the genetic intervals to which they have been mapped (see Additional data file 5). Using Perl scripts, we mapped every sequence on our arrays to the human genome using data from the UCSC genome browser [80]. Genes in the corresponding compartment expression set that were located in the genetic interval associated with each compartment-specific disease were identified. For the benchmark analysis of diseases that were associated with known genes, we also identified all genes in the human genome that fell into the genetic interval associated with each disease. The compartment expression sets and our lists of candidate genes for the 147 diseases we analyzed can be found in Additional data file 6.

## Additional data files

The following additional data are available with the online version of this paper. Additional data file 1 contains the step-by-step amplification protocol used in this work. Additional data file 2 is a table detailing RNA isolation and amplification yields. Additional data file 3 contains more detailed supplemental materials and methods. Additional data file 4 contains the compartment gene lists used in the disease gene analysis. Additional data file 5 contains the list of diseases for each compartment with their mapped genetic intervals. Additional data file 6 contains the results of the disease gene analysis, including the list of candidate genes for each disease.

## Supplementary Material

Additional data file 1 Step-by-step amplification protocol. Click here for file

Additional data file 2 A table detailing RNA isolation and amplification yields. Click here for file

Additional data file 3 Detailed supplemental materials and methods. Click here for file

Additional data file 4 Compartment gene lists used in the disease gene analysis. Click here for file

Additional data file 5 The list of diseases for each compartment with their mapped genetic intervals. Click here for file

Additional data file 6 Results of the disease gene analysis, including the list of candidate genes for each disease. Click here for file

## Figures and Tables

**Table 1:** Compartment gene sets are enriched for candidate genes of ocular diseases

	Disease-associated genes on array	Disease-associated genes expressed in affected compartment	Percentage of known disease-associated genes identified	Average fold enrichment compared to total number of genes in interval	*P*-value
Lens	15	8	53	2.4	0.002
Cornea	13	9	69	2.0	0.005
Retina	42	23	55	2.3	0.0004

Arrays were standardized to the same median intensity and genes exhibiting minimum intensities of 2,500 in any compartment were identified. Genetic diseases affecting the lens, cornea, or retina were collated from the RetNet [71] and Online Mendelian Inheritance in Man [72] databases, along with their cytogenetic map positions. The table indicates the number of cloned disease genes on the arrays, the number contained in a given compartment gene set, the percentage of known disease genes included in the signatures, the average fold enrichment compared to the total number of genes in each cytogenetic interval, and the statistical significance of this enrichment (using the hypergeometric distribution).
