# Supplementary material for: Differential gene expression in anatomical compartments of the human eye
Source: Genome Biol. 2005 Aug 17;6(9):R74. doi: 10.1186/gb-2005-6-9-r74 (PMC1242209; doi:10.1186/gb-2005-6-9-r74)
Supplement: Additional data file 1 — Step-by-step amplification protocol. [file gb-2005-6-9-r74-S1.doc]

# Additional File 1: Protocol for mRNA amplification and target preparation

# Based on Wang et. al., *Nature Biotechnology*, April, 2000

# Protocol written by Kate Rubins and Max Diehn

## Isolate Total RNA using Qiagen midi kit (Cat#75142) (see manufacturer's protocol) or by Trizol (Gibco BRL Cat# 15596-026) extraction (see manufacturer's protocol). Resuspend total RNA in DEPC water at 1ug/ul concentration.

Primer Sequences:

- oligo dT Primer= oligo dT(15)-T7 primer (5’ AAA CGA CGG CCA GTG AAT TGT AAT ACG ACT CAC TAT AGG CGC T(15) 3’)
- TS (template switch) oligo primer (5’ AAG CAG TGG TAA CAA CGC AGA GTA CGC GGG 3’)

All incubations are done in a thermal cycler. For starting amounts of less than or equal to 1ug of total RNA a second round of amplification may be required to generate enough aRNA for a microarray hybridization (2.5-3ug aRNA/hybridization.)

**First strand cDNA synthesis:**

In PCR reaction tube, mix

| Ammt | Reagent |
| --- | --- |
| 0.5-3ug | total RNA |
| 9ul | DEPC H2O |
| 1ul | (1ug/ul) oligo dT(15)-T7 primer |

70C for 3min, cool to room temperature.

Add to PCR Tube (separately or in a Master Mix)

| Ammt | Reagent |
| --- | --- |
| 4ul | 5X First strand buffer |
| 1ul | 1ug/ul TS (template switch) primer |
| 2ul | 0.1M DTT |
| 1ul | RNaseIN (Promega Cat# N2111) |
| 2ul | 10mM dNTP (Pharmacia Cat# 27-2035-02) |
| 2ul | Superscript II (Gibco BRL Cat# 18064-071) |

42C for 90min in thermal cycler.

(Note: buffer and 0.1M DTT come with SS II)

**Second strand synthesis:**

Add to PCR Tube

| Ammt | Reagent |
| --- | --- |
| 106 ul | DEPC H2O |
| 15ul | Advantage PCR buffer |
| 3ul | 10 mM dNTP mix |
| 1ul | RNase H (2U/ul Gibco BRL Cat# 18021-071) |
| 3ul | Advantage Polymerase (Clontech Cat# 8417-1) |

37C for 5min to digest mRNA, 94C for 2min to denature, 65C for 1min for specific priming and 75C for 30min for extension.

Stop reaction with 7.5ul 1M NaOH solution containing 2mM EDTA

Incubate at 65C for 10min to inactivate enzyme.

**DS cDNA cleanup:**

**Add to PCR Tube**

| Ammt | Reagent |
| --- | --- |
| 1ul | 0.1ug/ul Linear Acrylamide (Ambion Cat# 9520) |
| 150ul | Phenol: Chloroform: Isoamyl alcohol 25:24:1 (Boehringer Mannhem Cat #101001) |

Mix well by pipeting (be careful not to spill or contaminate).

Transfer the slurry solution to Phase lock gel tube (5’-3’ Inc. Cat# p1-257178)

Spin at 14,000rpm for 5min at room temperature.

Transfer the aqueous phase to RNase/DNase-free tube **(stopping point)**

Add 70ul of 7.5M ammonium acetate (Sigma Cat# A2706) and gently mix.

Add 1ml 100% room temperature ethanol.

Centrifuge at 14,000rpm for 20min at room temperature.

Prepare Bio-6 Chromatograph column (Bio-Rad Cat# 732-6222)

Shake well before draining to get rid of air bubbles - otherwise it drains very slowly! When opening column, sometimes you will observe gel in the underside of the cap. This should be aspirated off to prevent contamination.

Wash column three times with 700ul DEPC H2O and spin at 700xg for 2min at room temperature.

Remove flow-through. Make sure all liquid is drained out of column. Spin again at 700xg for 2min.

Wash pellet with 500ul 100% EtOH and spin pellet down at maximum speed for 6min.

Air dry pellet and resuspend ds cDNA in 60ul DEPC H2O.

Load 60ul sample to the center of the Bio-6 column and spin at 700xg for 4min. **(stopping point)**

Dry sample by speedvac and resuspend in 8ul DEPC water. **(stopping point)**

# In Vitro TRanscription

(Ambion; T7 Megascript Kit #1334)

In PCR reaction tube, mix

| Ammt | Reagent |
| --- | --- |
| 2 ul | of each 75mM NTP (A, G, C and UTP) |
| 2 ul | Reaction buffer |
| 2 ul | Enzyme mix (RNase inhibitor and T7 phage polymerase) |
| 8 ul | ds cDNA |

Incubate at 37C for 5-6hr.

**ARNA PURIFICATION USING QIAGEN RNEASY COLUMNS**

Make up RLT w/ â-ME and H2O Master Mix:

Per sample:

3.5ul β-ME

80 ul H2O

350ul RLT

Pre-aliquot 430ul RLT w/ β-ME and H2O to 1ml eppendorfs.

Transfer contents of in vitro transcription mix to the Rnase/Dnase-free tube.

Mix well. **(stopping point –80 overnight)**

Add 250ul ethanol (95%) and mix well by pipetting. (Do not spin here!)

Apply sample (700ul) to RNeasy mini spin column sitting in a collection tube. Centrifuge 15 sec at >= 8000 x g. Discard flow through.

Transfer RNeasy column to a new 2-ml collection tube (supplied). Add 500ul Buffer RPE (which must contain ethanol) and centrifuge 15 sec at >=8000 x g. Discard flow-through but re-use tube.

Pipet 500ul Buffer RPE onto RNeasy column and centrifuge for 2 min at maximum speed.

Remove flow through and pipet another 500ul Buffer RPE onto column. Centrifuge for 2 min at maximum speed. [This is an additional wash that is not in the Qiagen protocol which we have found necessary because of GITC contamination in the eluted RNA.]

Place RNeasy spin column into a new 1.5-ml or 2-ml collection tube (not supplied) and spin at full speed for 1 min to completely dry column.

Transfer RNeasy column into a new 1.5-ml collection tube (supplied) and add 30ul RNase-free water directly onto membrane. Centrifuge for 1 min at >=8000 x g to elute. Repeat if expected yield is >= 30ug.

Check RNA concentration and quality by measuring OD260 and OD260/280.

**Target labeling by reverse transcription**

Follow standard microarray labeling techniques, using between 3-6ug of aRNA as input. Remember to use dN6 primer instead of oligo-dT. Use between 5-10ug of primer.
