# Supplementary material for: Differential gene expression in anatomical compartments of the human eye
Source: Genome Biol. 2005 Aug 17;6(9):R74. doi: 10.1186/gb-2005-6-9-r74 (PMC1242209; doi:10.1186/gb-2005-6-9-r74)
Supplement: Additional data file 2 — A table detailing RNA isolation and amplification yields. [file gb-2005-6-9-r74-S2.doc]

**Additional File 2: Amplification details**

| **Globe** | **Mass of total RNA (ug)** | **Total RNA input to amplification reaction (ug)** | **Mass of aRNA (ug)** | **Fold amplification of mRNA** |
| --- | --- | --- | --- | --- |
| G2 nasal retina | 6.2 | 6.0 | 11.4 | 76 |
| G2 inferior retina | 7.2 | 7.0 | 15.9 | 91 |
| G2 superior retina | 15.0 | 5.4 | 10.5 | 78 |
| G2 macula | 12.0 | 6.0 | 16.2 | 108 |
| G7 macula | 4.6 | 4.6 | 16.5 | 144 |
| G5 macula | 2.2 | 2.2 | 20.5 | 373 |
| G3 macula | 16.7 | 5.0 | 32.0 | 256 |
| G3 superior retina | 62.5 | 4.0 | 30.2 | 302 |
| G3 nasal retina | 29.8 | 5.0 | 36.0 | 288 |
| G3 temporal retina | 17.4 | 5.0 | 40.5 | 324 |
| G5 nasal retina | 9.1 | 5.0 | 40.5 | 324 |
| G5 temporal retina | 17.2 | 5.0 | 32.5 | 260 |
| G8 retina | 18.4 | 5.0 | 15.8 | 126 |
| G7 nasal retina | 22.3 | 5.0 | 22.2 | 178 |
| G7 temporal retina | 13.8 | 5.0 | 29.1 | 233 |
| G1 retina | 81.0 | 4.0 | 8.1 | 81 |
| G2 optic nerve | 2.0 | 2.0 | 3.6 | 74 |
| G7 optic nerve | 17.4 | 5.0 | 5.7 | 46 |
| G6 optic nerve | 14.0 | 5.0 | 4.6 | 36 |
| G4 optic nerve | 12.7 | 5.0 | 8.6 | 68 |
| G1 optic nerve | 8.4 | 5.0 | 7.2 | 58 |
| G4 cornea | 3.1 | 3.1 | 13.7 | 177 |
| G5 cornea | 6.1 | 5.0 | 12.0 | 96 |
| G6 cornea | 9.0 | 5.0 | 3.4 | 27 |
| G7 cornea | 7.2 | 5.0 | 11.4 | 91 |
| G6 ciliary body | 14.1 | 5.0 | 3.4 | 27 |
| G5 ciliary body | 47.3 | 5.0 | 2.3 | 18 |
| G3 ciliary body | 53.2 | 4.7 | 10.8 | 92 |
| G3 iris | 12.7 | 5.0 | 2.7 | 22 |
| G6 iris | 7.0 | 5.0 | 3.0 | 24 |
| G1 ciliary body | 15.0 | 4.0 | 4.2 | 42 |
| G1 lens | 3.0 | 3.0 | 5.4 | 72 |
| G5 lens | 1.1 | 1.1 | 5.7 | 207 |
| G4 lens | 1.2 | 1.2 | 4.0 | 133 |
| G6 lens | 3.1 | 3.1 | 4.6 | 59 |

Note: In order to calculate “fold amplification,” we estimated that 2.5% of the input total RNA was composed of mRNA.
