# Supplementary material for: Differential gene expression in anatomical compartments of the human eye
Source: Genome Biol. 2005 Aug 17;6(9):R74. doi: 10.1186/gb-2005-6-9-r74 (PMC1242209; doi:10.1186/gb-2005-6-9-r74)
Supplement: Additional data file 3 — Detailed supplemental materials and methods. [file gb-2005-6-9-r74-S3.doc]

## Additional File 3: Supplemental Materials and Methods

**Tissue Specimens**. Eight whole globes (G1-G8) were harvested from autopsy donors (age range=30-85 years-old) within 24 hours of death, and the tissues were immediately stored at 4^◦^ C in RNAlater (Ambion). Four of the globes were from female donors (G3, G6-8) and four were from male donors (G1, 2, 4, 5). Globes 4 and 5 were harvested as a set from a single donor, as were globes 6 and 7. No ophthalmologic clinical records were available for any of the globes at the time of harvest. Seven of the globes (G1-G7) were dissected into the following components: cornea, lens, iris, ciliary body, retina, and optic nerve, while only retinal tissue was available from G8. The maculas and the peripheral retinal tissues were further dissected from several of the retinal samples. The macula was defined as the visible xanthophyll-containing tissue temporal to the optic nerve, which encompassed an approximate area of 4 mm^2^. For comparison purposes, three post-mortem brain specimens were analyzed.

# **RNA extraction and amplification**. Specimens were disrupted in TRIZOL (Gibco) solution using a tissue homogenizer. Samples were processed according to the manufacturer’s protocol until the aqueous supernatant was retrieved. The supernatant was mixed with 1 volume of 70% ethanol, applied to an RNeasy column (Qiagen), and purified according to the manufacturer’s protocol. RNA quality and quantity were assessed by gel electrophoresis and spectrophotometer measurements. Total RNA was amplified using a single round, linear amplification method [9]. Tissue samples that yielded inadequate amounts of RNA were excluded from any further analysis. A reference mixture of mRNAs derived from 10 different cell lines (Universal Human Reference RNA, Stratagene) was used in all experiments as an internal standard for comparative 2-color fluorescence hybridization.

**Microarray Procedures**. Human cDNA microarray construction and hybridization were as previously described [12]. The microarrays contained 43,198 elements, representing ~30,000 genes (estimated by UniGene clusters) and were manufactured by the Stanford Functional Genomics Facility (http://www.microarray.org). In each analysis, amplified RNA from an eye tissue sample was labeled with Cy5, and amplified reference RNA was labeled with Cy3. The two labeled samples were combined, and the mixture was hybridized to a microarray. Arrays were scanned using a GenePix 4000B scanner (Axon Instruments Inc.). The array images were processed using GenePix Pro 3.0, and the resulting data were indexed in the Stanford Microarray Database (SMD) and normalized using SMD’s default total intensity normalization algorithm. Searchable figures and all raw microarray data can be found at http://microarray-pubs.stanford.edu/eyecompartments.

**Normalization Procedure.** Prior to further analyses, the microarray data were normalized using SMD’s default total intensity normalization algorithm. Briefly, the goal of normalization is to remove systematic biases from microarray data, such as dye effects and scanner variation, so that multiple microarrays can be compared to each other for analyses such as hierarchical clustering. Total intensity normalization ensures that the average log ratio on every array is zero, thus removing potential systematic biases (such as one channel having been scanned at higher sensitivity settings). The normalization constant is computed based on all non-flagged data meeting a quality filter. This is determined using a threshold value of the percentage of spot pixels with intensities more than one standard deviation above the background in the Cy3 and Cy5 channels. The threshold value is initially set to > 0.65. If fewer than 10% of the elements on an array pass these criteria, the threshold is relaxed by 0.05 until 10% of elements are included or 0.55 is reached. The normalization constant is calculated using these spots and is then applied multiplicatively to all array elements, regardless of whether they were used in the calculation. More in-depth discussion of this procedure can be found at SMD (http://genome-www5.stanford.edu/help/results_normalization.shtml).

**Bioinformatic Analyses.** For the data shown in Figures 1 and 2, only elements for which at least 50% of the measurements across all samples had fluorescence intensity in either channel at least 3.25-fold over background intensity were included. The logarithm of the ratio of background-subtracted Cy5 fluorescence to background-subtracted Cy3 fluorescence was calculated. Then values for each array and each gene were median centered, and only cDNA array elements for which at least two measurements differed by more than 2.5-fold from the median were included in subsequent analyses. For the data in Figure 3, we employed the Statistical Analysis of Microarrays (SAM) package [13]. Only elements for which the intensity to background ratio was at least 3.25 in at least 35% of the retina samples were considered. Only genes whose expression significantly differed between the macula and peripheral retina (false discovery rate < 0.05 with 500 permutations) were selected. Finally, in order to focus on genes with the largest absolute difference in expression between the two regions, we selected genes whose expression differed by at least 4-fold from the median in at least 2 samples.

**Candidate disease gene analysis.** To identify the gene sets expressed in each compartment, background-subtracted Cy5 intensities from each microarray were standardized to an array-median of 1,500, and genes exhibiting an average intensity of at least 2,500 in a compartment were identified (see Additional File 4). This threshold was chosen empirically for several reasons. First, using the 2,500 cut-off resulted in greater than 85% of the retinal signature from Figure 1 to be included in the retina set (i.e. the genes specifically expressed in retinal tissues), while less than 5% of these genes were contained in any of the other compartment gene sets (as defined in Figure 1). Second, using the 2,500 intensity cut-off, an average of ~12% of the ~40,000 elements on our arrays was included in each compartment list. This percentage rose steeply to ~50% when we tested an intensity cut-off of 1,000. Thus, relaxation of the intensity threshold would have resulted in a significantly lower enrichment factor, as many more genes mapping to each disease interval would have been included in the compartment expression lists. We chose the intensity approach over simply selecting genes that there were selectively expressed in each compartment because we did not want to exclude possible candidate genes that were expressed in more than one compartment but that caused disease in only one of them.

Genetic diseases affecting the lens, cornea, or retina were collated from OMIM [14] and the Retinal Information Network (Retnet: http://www.sph.uth.tmc.edu/RetNet/home.htm), along with the genetic intervals to which they have been mapped (see Additional File 5). Using Perl scripts, we mapped every sequence on our arrays to the human genome using data from the UCSC genome browser (http://genome.ucsc.edu/). Genes in the corresponding compartment expression set that were located in the genetic interval associated with each compartment-specific disease were identified. For the benchmark analysis of diseases that were associated with known genes, we also identified all genes in the human genome that fell into the genetic interval associated with each disease. The compartment expression sets and our lists of candidate genes for the 147 diseases we analyzed can be found in Additional File 6.
